# Modeling population heterogeneity from microbial communities to immune response in cells

**DOI:** 10.1007/s00018-019-03378-w

**Published:** 2019-11-25

**Authors:** Tal Pecht, Anna C. Aschenbrenner, Thomas Ulas, Antonella Succurro

**Affiliations:** 1 Genomics and Immunoregulation, Life and Medical Sciences (LIMES) Institute, University of Bonn, Bonn, Germany; 2 Department of Internal Medicine and Radboud Center for Infectious Diseases (RCI), Radboud University Medical Center, 6525 Nijmegen, The Netherlands; 3 West German Genome Center (WGGC), University of Bonn, Bonn, Germany

**Keywords:** Population heterogeneity, Computational modeling, Systems biology, Microbial communities, Immune response

## Abstract

Heterogeneity is universally observed in all natural systems and across multiple scales. Understanding population heterogeneity is an intriguing and attractive topic of research in different disciplines, including microbiology and immunology. Microbes and mammalian immune cells present obviously rather different system-specific biological features. Nevertheless, as typically occurs in science, similar methods can be used to study both types of cells. This is particularly true for mathematical modeling, in which key features of a system are translated into algorithms to challenge our mechanistic understanding of the underlying biology. In this review, we first present a broad overview of the experimental developments that allowed observing heterogeneity at the single cell level. We then highlight how this “data revolution” requires the parallel advancement of algorithms and computing infrastructure for data processing and analysis, and finally present representative examples of computational models of population heterogeneity, from microbial communities to immune response in cells.

## Introduction

The concept of heterogeneity is a universal and a key feature of nearly all natural systems. In biology, heterogeneity has been documented since a long time, as early as 1957, with Novick and Weiner describing high stochasticity in gene expression levels of a bacterial population [1]. It is now clear that both single-celled and multicellular organisms may benefit from preserving heterogeneity between individuals of the population, and variability in the phenotype of immune cells has been shown to provide on average robust response to infections. For instance, an early expression of a set of antiviral genes in few “precocious” cells within a population of dendritic cells was shown to drive a population-level response at later timepoints, orchestrating a complex, coordinated response within the cellular population through cell-to-cell communication [2].

**Fig. 1:**
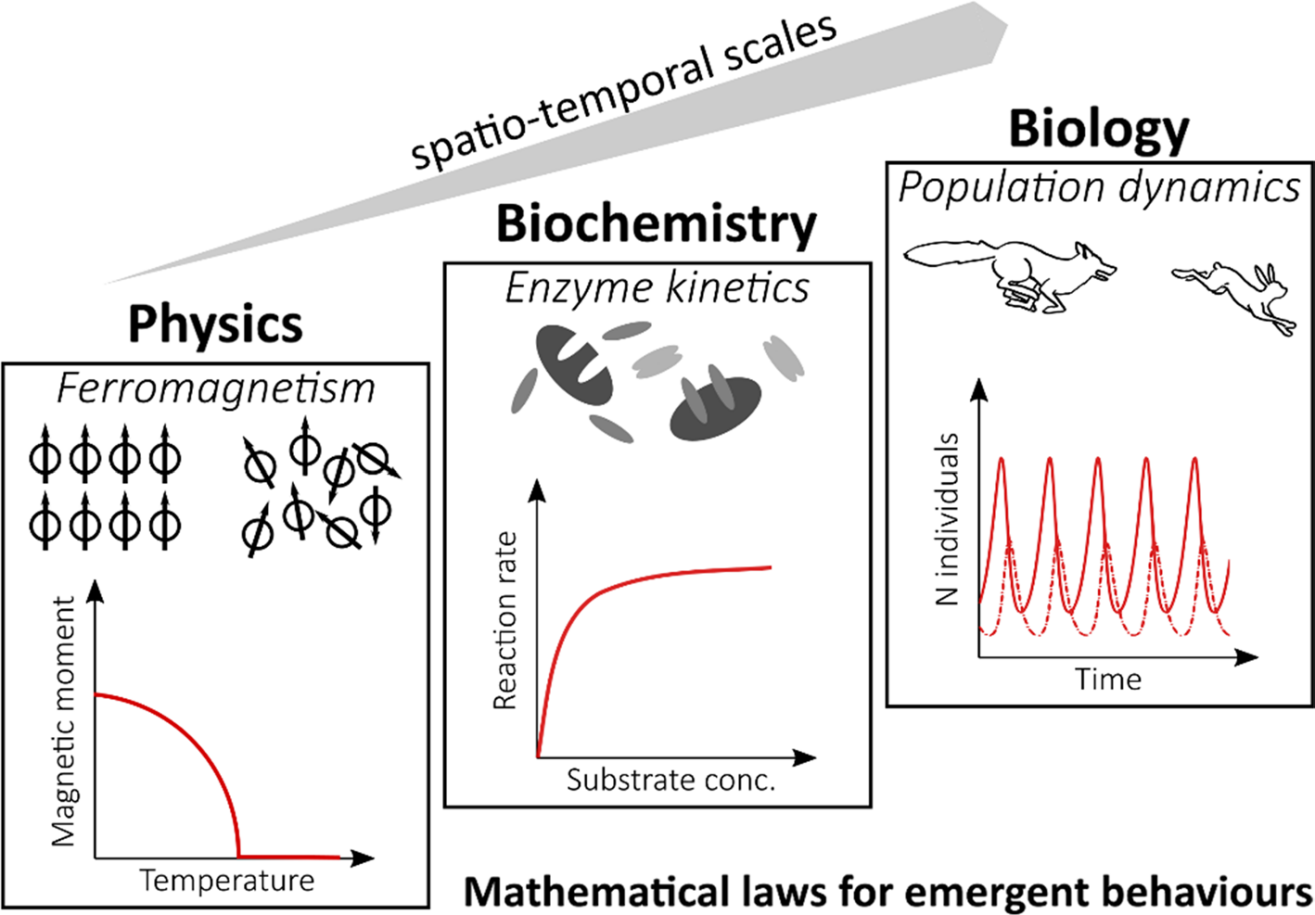
Emergent properties across scales and disciplines. Systems with different typical spatio-temporal scales can in general be described either at the micro-scale (describing in details the individual interactions of the system components) or at the macro-scale (capturing the emergent behavior of such interactions with simpler mathematical models. For example, in ferromagnetic materials the magnetic moment derives from the alignment of individual electrons dipole moments. At temperatures above a critical point (Curie temperature), entropy disrupts such alignment and the material is no more magnetic. Moving from electrons to molecules and proteins, enzymes convert substrates into products after binding the molecules. The reaction requires different steps to successfully happen, but the product formation rate can be well described as a function of substrate concentration, e.g., by the Michaelis–Menten equation. Finally, to describe populations of organisms’ general growth, laws can be defined that capture the overall trends resulting from individual-to-individual interactions. In predator–prey models, few differential equations describe how the different populations harm or benefit each other

**Fig. 2:**
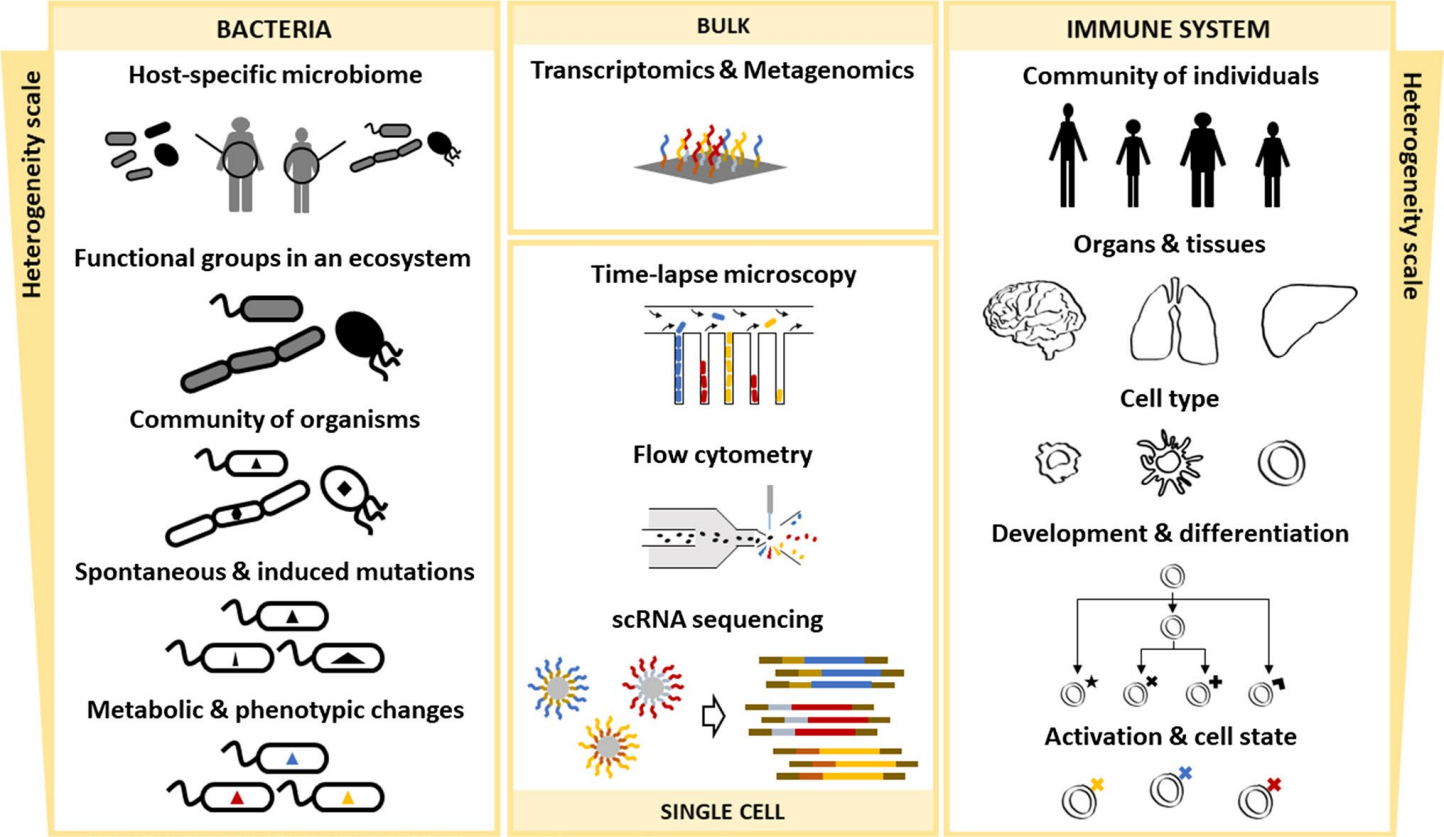
Heterogeneity and experimental methods across spatio-temporal scales and biological systems. Heterogeneity for bacterial communities can be found in ecosystems composed of distinct bacteria species, which usually specialize to occupy different metabolic niches and can organize into functional groups. Monocultures can exhibit heterogeneity at different temporal scales when mutations generate subpopulations, and within isogenic populations phenotypic and metabolic heterogeneity can be observed. Heterogeneity in the context of the immune system presents additional degrees of complexity, as it is a characteristic of multicellular eukaryotes. From individuals within a population to organs within an individual, even finer scales of heterogeneity can be observed at the cell type level (e.g., T cells, dendritic cells, and macrophages). Cells exhibit further heterogeneity as they develop and undergo differentiation and, in addition, can be observed in different activation states with different phenotypic profiles. Despite the completely different biology, similar experimental methods can be used to investigate heterogeneity at different scales. From meta-omics bulk techniques to microfluidic growth chambers monitored with time-lapse microscopy, to flow cytometry, to the latest advancements in RNA sequencing of single cells

Stochasticity is universally observed in the natural sciences and can be considered an intrinsic property of physical systems, from elementary particle interactions to gene expression. Indeed, in many systems, events happening at a fundamental scale (where randomness is typical and often essential to describe the system) give rise to collective, emergent behaviors that can be observed and described at a macroscopic scale. Figure 1 illustrates few examples from different disciplines with different spatio-temporal scales, where general laws can describe emergent properties of the system such as population oscillations in Lotka–Volterra predator–prey models. The apparent uniformity at the macroscopic scale, however, is just the result of summing up the microscopic heterogeneity of the components of the system, meaning that the overall differences in individual features average out in a sufficiently large population. In this review, we will focus on the heterogeneity observed in two kinds of settings: bacterial cultures and the immune system (Fig. 2). Despite the evident biological difference between bacteria and mammalian immune cells, heterogeneity can be investigated in similar ways, and cross-disciplinary methods (experimental and theoretical) can provide essential insights. A recent review by Jolly et al., for example, drew a parallel between bacteria and cancer cells in the context of phenotypic plasticity [3]. Stochastic differentiation (also known as “bet-hedging”) in clonal bacterial population means that subpopulations with different phenotypes can randomly appear, thus providing a selective advantage to survive in fluctuating and unpredictable environments. This kind of non-genetic heterogeneity, where cells can switch to different phenotypic states without altering their genomes (either randomly or in response to changes in the environment) is extremely relevant in microbiology, as it is one of the mechanisms by which bacteria can survive antibiotic treatments [4]. Indeed, when faced with high drug doses, bacteria can develop resistance (through inheritable genetic mutations) or persistence (through phenotypic plasticity). In the latter case, the surviving population will return to a state similar to the pre-treatment one once the environment normalizes. Recently, similar mechanisms have been observed in cancer cells, offering the perspective that abstracted models to study heterogeneity can have high cross-disciplinary potential for knowledge transfer [3].

Nowadays, computational biology is a well-established field rapidly gaining visibility and interest from the experimental community. Besides the widely recognized applications in data analysis (bioinformatics), computational biology encompasses also the more theoretical discipline of mathematical modeling. Mathematics has been used for centuries in ecology to describe, e.g., population dynamics [5]. Over the last few decades an increased interest in challenging the mechanistic understanding of biological systems, paired with technological advances providing high-quality quantitative biological data, allowed the field of theoretical biology to flourish, with applications ranging from metabolic engineering [6] to personalized medicine [7]. The fundamental concept behind the art (and power) of theoretical approaches is abstraction. Indeed, models can be constructed including all possible minute details of the system, in this way drawing a complete picture of the system according to the state-of-the-art knowledge, but such over-representation might hinder their predictive power, especially when model parameters cannot be directly measured or derived from first principles and have to be purely fitted to data, which might lead to overfitting [8]. Instead, by selecting only the relevant features of a system and translating them into a mathematical model, it becomes possible not only to gain insights on fundamental mechanisms, but also to generalize the model to different systems sharing the same characteristics. While we do not have yet a formal theory of cellular heterogeneity, methods from statistical physics and information theory can be combined with the latest omics technologies to describe non-equilibrium biological systems [9].

Over the past years, the fields of microbiology and immunology have shown fast-paced advances, also regarding the development of theoretical approaches [10, 11]. In both these fields, the role of cellular heterogeneity has become increasingly relevant, also thanks to technological advancements such as single cell RNA sequencing (scRNA-seq) [12]. With new high-throughput and high-resolution data available for training and parameterization, the potential of computational models to obtain predictive results and understand fundamental biological mechanisms can be redefined. However, with big data come big responsibilities, and a number of bioinformatics challenges. In this review, we will first briefly present an overview of the experimental developments (with the corresponding data analysis challenges) that allowed quantitative observations of cellular heterogeneity. We will then discuss the possible heterogeneity levels in bacterial populations and the immune system (Fig. 2), highlighting examples how cellular diversity can be observed at the different levels. Finally, we will introduce mathematical modeling and present how it has helped to understand and predict cellular behavior.

### Experimental advances and challenges: dealing with big data and resolving bulk measurements

Differences in cellular phenotype giving rise to heterogeneity can originate from stochastic changes in gene and protein expression, from the effect of regulatory pathways such as epigenetic and post-transcriptional/translational modifications, and from the activity of signaling and metabolic pathways [12, 13]. Until experimental techniques developed to offer quantitative insights into such variations at high resolution, cellular heterogeneity could not be truly appreciated. As illustrated in Fig. 2, different levels of “heterogeneity” can be defined based on the scales at which the system is to be investigated. To resolve these different scales, new experimental methods had to be developed. Changes in protein expression were already recognizable with traditional methods in the life sciences, for example, microscopy or flow cytometry, which allow single cell resolution [14]. With the addition of lasers and input channels to multicolor flow cytometry instruments and with the discovery and development of fluorophores, parallel detection in more channels was enabled. Furthermore, improvements of microscopes and down-sizing instruments dimensions made these technologies more accessible and affordable for many labs [15]. Furthermore, the ability to sort out specific cellular populations using fluorescence-activated cell sorter (FACS), enabled the downstream analysis of specific subclasses, and later on of single cells of interest [16]. An additional antibody-based platform for single-cell analysis is cytometry by time of flight (CyTOF), which allows the simultaneous measurement of more than 40 parameters of an individual cell [17]. Moreover, the development of better cameras and automated equipment for microscopes enabled time-lapse microscopy and automated high-content imaging and thus permitted both temporal and spatial resolution of biological processes [18]. Such methodological advancements were, for example, utilized for subcellular localization of more than 12,000 proteins detected by antibody-based immunofluorescence confocal microscopy [19].

With the improvement of these techniques over time, a higher resolution could be achieved with a larger repertoire of proteins identified at higher accuracy. Revealing heterogeneity at the transcriptional and epigenetic levels followed advancements in development of genome-wide analyses and more advanced Next-Generation Sequencing (NGS), also known as high-throughput sequencing, which includes a number of different modern sequencing technologies [20]. NGS technologies enabled the sequencing of RNA and DNA in a cost and time-effective manner, revolutionizing the ability of scientists to dissect the immune system repertoire [21]. Today, sequencing and analysis of a complete human genome can be done for less than $1000, and it is possible that by 2025, we will have sequenced up to 2 billion human genomes, thus covering a big proportion of world-wide genetic heterogeneity between individuals [22]. In microbiology, 16S ribosomal RNA (rRNA) gene amplicon sequencing has been widely used to profile a bacterial community composition. More recently, shotgun metagenomics enabled the sequencing of the full genome of all organisms present in a sample, providing information on functional roles as well [23]. Furthermore, developments in high-throughput genetic screenings such as mariner transposon insertion sequencing (INSeq) have enabled the systematic study of gene essentiality and function in bacterial populations [24]. With improvement of single-cell RNA preparation [25], and microfluidics devices [26], NGS has been applied to single cell measurements, such as scRNA-seq [27]. Single-cell omics technologies such as scRNA-seq will play key roles in untangling the complex and diverse composition of phenotypes within cellular populations, as extensively described by recent review articles [28, 29]. Single-cell epigenomics measurements may capture modifications such as DNA methylation, histone modifications (e.g., methylation and acetylation) [30] and chromatin accessibility at the single cell resolution [31]. Furthermore, improvement in sample preparation makes it possible to yield multiple measurements from individual samples or even individual cells, presenting opportunities to simultaneously measuring single cell transcriptomics and epigenomics information [32]. Efforts in single cell data generation from human samples are united under the Human Cell Atlas project, an exemplary international collaboration that aims to characterize all human cell types, including immune cells, on the molecular (e.g., gene and protein expression) and descriptive (e.g., location and morphology) level in health and disease [33].

Besides its scientific mission to collect comprehensive biological data, the Human Cell Atlas also aims to address technical issues in data generation, such as batch effects and variations in the techniques and protocols being used. Indeed, the constantly increasing possibility to produce novel data requires standardized common rules for data generation and analysis. For example, the NGS community needed to adapt the processing pipelines to speed up the process of alignment, which is a way of mapping the sequences or reads to a reference sequence representing mostly the entire genome of the organism of interest. Novel approaches such as pseudoalignments (see examples in Table 1) were able to accelerate the alignment process by two orders of magnitude with respect to earlier methods and achieve comparable precision. In parallel and directly correlated to improving the processing speed, comes the need to cope with the increased amount of data. Indeed, acquiring, storing, distributing, and analyzing such large amounts of data require massive and unique technological solutions. Today, computing power is increased with high-performance computing (HPC) cluster services that can provide the necessary central processing unit (CPU) and graphics-processing unit (GPU) power required to deal with genomic data. However, with growing amounts of data, many present algorithms are not scaling well and computational memory, rather than CPUs or GPUs, becomes a performance bottleneck. A promising solution could be the usage of novel computer hardware architecture such as memory-driven computing combined with optimized genomics data algorithms, which was shown to accelerate pseudoalignment with Kallisto by more than two orders of magnitude with identical accuracy [34].

**Table 1:** Developments in bioinformatics tools are fast-paced

Purpose	Pseudoalignment	Reproducibility	Guided bulk RNA-seq analysis	Guided scRNA-seq analysis	scRNA-seq command line	Batch correction in bulk RNA-seq	Batch correction in scRNA-seq
Tools and algorithms	Kallisto [105]	Snakemake [106]	START [107]	ASAP [108],	SINCERA [109]	Limma [110]	Canonical correlation analysis [111]
	Salmon [112]	Docker [113]	DEBrowser [114]	FastGenomics [115]	Seurat [111]	ComBat [116]	
	Sailfish [117]	Singularity [118]	iDEP [119]	Granatum [120] and GranatumX [121]	Scanpy [122]	SVA [123]	Mutual nearest neighbors [124]
			Shiny-Seq [125]	Monocle [126]			

Here we list some of the latest bioinformatics methods applied for RNA analysis, from sequence alignment to downstream data analysis

Experimental approaches and the differences in the protocols on sample handling and preparation are important considerations when trying to ensure reproducibility and comparability of data. Beyond the technical aspects of data generation and the critical question on uncertainty quantification, computational reproducibility (being able to perform the same processing or analysis on another system or at a later timepoint and getting the same results [35]) is a principle of scientific methodology that—unfortunately nowadays—has been too often overlooked in computational analysis [36]. A very promising approach to achieve reproducibility for data analysis is to use combinations of workflow management systems and land container (Table 1). A container is a unit of software that packages up code and all its dependencies, so that the application runs reliably on other systems. Using a workflow management system, reproducible pipelines can be easily scaled in server, cluster, and cloud environments. The combination of both allows compiling complex workflows, with all necessary tools in the respective environment and version stored as container (images) in an online database. In addition, algorithms and workflows with convenient and intuitive interfaces are critical for non-specialists to process and analyze omics data coming from either bulk or single cell experiments. For example, interactive and intuitive web based applications (Table 1) were designed to assist experimental biologists to explore, visualize, and interpret bulk RNA-seq data. For scRNA-seq data, there are platforms that provide an intuitive graphical user interface based partially or completely on well-known command-line tools (Table 1). Despite the growing availability of standardized analysis tools, reproducibility can still be hindered by technical heterogeneity (batch effects) due to aspects such as different times of experiments, different experimenters, reagent lots, sequencing technology, among others. Resolving batch effects is a challenging task. For bulk RNA-seq data, there are methods, which can account for known or even unknown technical heterogeneity (Table 1). For single-cell analysis, correction for technical heterogeneity is even more challenging and from bulk RNA-seq known algorithms are not directly applicable to single cell data. However, recent reports introduced the use of new methods (Table 1) to successfully correct batch effects in scRNA-seq data. Especially, the newly introduced anchoring enables to integrate single-cell data not only across different single cell sequencing technologies, but also across other omics technologies [37]. Nevertheless, there is always a certain risk when using algorithms for correction of technical heterogeneity. Choosing an unsuitable algorithm or the improper application of such can lead to overcorrection of the data or reduction of unwanted technical and concomitantly biological heterogeneity, which should be reserved for further downstream analysis.

Cellular heterogeneity can be studied at the genomic, epigenomic, transcriptomic, proteomic, and metabolic cellular level. Single cell experiments perform measurements on the individual cells, in contrast to bulk experiments that are done on ensembles of cells and, therefore, return only the average characteristics of the population. Nonetheless, new analysis methods allow the deconvolution of cellular heterogeneity from bulk data. For example, for bulk transcriptomics data, there are approaches using known gene expression signatures for the deconvolution of cell types [38]. Monaco et al. recently developed a method to study the cell composition of bulk transcriptomics data (both RNA-seq and microarray) generated from peripheral blood mononuclear cells, which contain populations of lymphocytes, myeloid and to a lesser degree granulocyte and progenitor cells [39]. The authors identified sets of cell type-specific, co-expressed, and housekeeping genes across the 29 cell types, which were then used for accurate deconvolution applying robust fitting of linear models.

As mentioned earlier, the constantly increasing accessibility of NGS methods makes the generation of omics data easier and less expensive. Today’s single cell NGS technologies can generate an enormous amount of data, where noise, e.g., from amplification and dropout is a common problem. Kharchenko et al. proposed a noise tolerant Bayesian approach, which allows the identification of differential gene expression and subpopulations in single-cell data using a probabilistic model of expression-magnitude distortions [40]. Recently, Sun et al. presented a Bayesian mixture model for single cell sequencing accounting for both individual and technical heterogeneity for improved clustering of scRNA-seq data [41]. With the advent of machine learning methods, more powerful algorithms can be applied to analyze single-cell data. For example, Eraslan et al. recently proposed an unsupervised neural network method (deep count autoencoder network) to reduce noise in sparse scRNA-seq data which scales linearly with the size of the data [42].

### Observing heterogeneity in bacterial populations

Heterogeneity in bacterial populations is observed at different scales (Fig. 2). An obvious example is heterogeneity in bacterial communities, where different species sharing the same environment can cooperate, compete for resources or neutrally co-exist. Such heterogeneity scale could be easily observed since the early years of microbiology, for example, by distinguishing cells under a microscope, but only with the advent of sequencing technologies, it became possible to systematically quantify the population dynamics in microbial consortia. With meta-omics approaches such as metagenomics and metatranscriptomics, the structure, ecological function, and collective metabolic potential of a bacterial community can be studied [43]. In general, these analyses can cover the broader levels of community heterogeneity often simultaneously, if the sequencing resolution allows (Fig. 2, left top three images), assigning an ecological or metabolic function by clustering genes shared among different strains, and identifying the individual genomes to assess the detailed composition of a community. Particular interest, especially during the last decade, has been addressed to understand the microbiome, i.e., the community of microbes living in association with a multicellular organism. Earlier studies relied on 16S rRNA gene amplicon sequencing to profile the microbiome phylogeny and identified relevant microbiome covariates from phenotypic data [44]. More recently, full metagenome analyses focused on characterizing host–microbiome interactions. To study host-dependent heterogeneity, in an impressive effort, Pasolli et al. sequenced over 150,000 genomes from about 10,000 body-wide human microbiome metagenomes, revealing a large fraction of previously unknown species [45]. The analysis of species-level genome bins of the gut microbiome revealed profound differences at the functional level for Western and non-Western lifestyles, pinpointing also microbes unique to specific environments. Focusing on functional roles, Levy et al. analyzed almost 4000 genomes of soil, root-associated, plant-associated, and non-plant-associated bacteria to identify gene sets relevant for plant colonization and for microbe–microbe competition [46]. Furthermore, a fairly advanced research field is the inference of community dynamics from time series of metagenomics data [47].

Bacteria and microbes in general are extremely diverse, and one could argue that heterogeneity is exhibited also within the same species (Fig. 2, left third image from the top). Indeed, because of the plasticity of their genome and short cell-division times coupled with random mutation events, prokaryotes can rapidly evolve and adapt to new environments. Such differentiation events leading to increasing divergences in the genome of an individual species are extremely relevant when studied in the evolutionary and ecological context. For example, Bhaya et al. used comparative genomics and metagenomics to assess the functional diversity of *Synechococcus* cyanobacteria sampled in microbial mat communities from Yellowstone hot springs [48]. Their analysis, comparing two isolates dominating environments of different temperatures and light, identified significant divergences in phosphate and nitrogen utilization pathways, and pointed to the possibility of recent and recurrent gene loss and gain of a urease cluster within the *Synechococcus* populations of the mat.

Until recently, cellular growth, genome adaptation, and gene expression in response to environmental changes have been measured mostly with bulk techniques. With the advent of single-cell methods, a deeper scale of bacterial heterogeneity was then revealed (Fig. 2, left bottom image). Indeed, monoclonal and isogenic populations can also exhibit heterogeneity at the level of gene expression and metabolic activity [49]. Microfluidic devices allow to isolate and track single bacterial cells, and in combination with fluorescent markers for gene expression and time-lapse microscopy, it is also possible to follow subpopulation dynamics in great detail. For example, in the “Mother machine” microfluidic chemostat (depicted in Fig. 2), a single mother cell is trapped into a closed channel and upon division the cells are pushed out into the feeding channel and get flushed away. With such device, it is possible to highly control the growth environment and measure precisely cell growth rates. Rosenthal et al. used the Mother machine to study the switch between two subpopulations of *Bacillus subtilis* marked with fluorescent promoters for key genes of the metabolic TCA cycle [50]. The authors set off to explore metabolic specialization in *B. subtilis* monoclonal cultures to understand the mechanism by which it switches from consuming glucose and malate and secreting acetate (which, being a weak organic acid, at high concentration becomes toxic for the cells) to consuming acetate and producing acetoin (a non-toxic pH-neutral metabolite). By quantitative single-cell fluorescence microscopy the authors observed that the genes encoding succinase co-A ligase (*sucC*, related to the TCA cycle activity) and acetolactate synthase (*alsS*, known to be involved in acetoin production) were most heterogeneously expressed which lead them to study these two subpopulations in more detail. Performing a time course analysis of the fraction of *sucC *+ and *alsS *+ cells, the authors observed that the subpopulation emergence coincided with the time at which acetate was produced and converted into acetoin, respectively, suggesting a previously unknown role for *sucC* expression in acetate production. Rosenthal et al. went deeper into the regulation of gene expression of the *sucC *+ subpopulation using FACS to isolate cells at the time of maximum acetate concentration and determined gene expression profiles by RNA-seq. Their analysis revealed a strong correlation in the expression of *sucC* and competence genes, which are overlapping with those genes involved in the switch of *B. subtilis* into the competent state, i.e., the cellular state, where the bacterium can transform by uptaking extracellular DNA. Finally, they followed the cell switch into the competence state with the Mother machine and measured the rates of transition between the *sucC *+ and *alsS *+ states, testing different medium composition to prove the environmental effect on gene expression. Collectively, their results point to an evolutionary advantage of metabolite-mediated metabolic switching in predictable environments (secretion and accumulation of by-products), in contrast to stochastic ‘bet-hedging’, a random or concerted phenotypic change that despite lowering fitness in favorable external conditions allows a population to survive unexpected challenges [51].

### Observing heterogeneity in the immune system

Similar to bacteria, multicellular organisms present vast biological heterogeneity, though moving from uni-cellular to multicellular organisms is not completely comparable, and additional levels that extend beyond the cellular and subcellular–molecular levels should be considered. Heterogeneity in the immune system has received much attention in recent years, especially due to technological and analytical advancements, as discussed previously and recently reviewed [52]. The immune system is a network of cellular (immune cells) and soluble components (i.e., cytokines, chemokines, antibodies, complement factors, and growth factors), acting throughout the body, performing tasks related to tissue maintenance, surveillance, repair, and defense against pathogens or cellular stress during acute and chronic challenges which can lead to pathological responses resulting in diseases. Already when exploring heterogeneity at the highest scale, i.e., the population-level, the immune system is highly diverse among different individuals [53]. Indeed, when moving from bench to bedside (i.e., from basic sciences to the clinic), on one hand patient-to-patient variability challenges the understanding of the immune system (Fig. 2, right top image), but on the other side, it guides the efforts towards the development of personalized and precision medicine [53]. On the scale of the individual, the immune system is highly heterogeneous across different organs and tissues (Fig. 2, right second image from top). Resident immune cells develop locally within each tissue (not necessarily sharing the same origin), adapt to a specific tissue microenvironment, and play distinct roles in specific organ and tissue functions. On the cellular and subcellular scale, inter-cellular heterogeneity is extremely broad, facilitating a flexible response of the immune system. Various classes of specialized and well-defined immune cell types exist including, e.g., macrophages, T cells, B cells, or dendritic cells, (Fig. 2, right middle image). Due to continuous production of immune cells from hematopoietic stem cells, precursors in many different developmental and differentiation states also exist, thereby significantly increasing cellular heterogeneity [54]. Furthermore, during any kind of challenge, additional immune cell states occur due to activation and differentiation processes (Fig. 2, right second image from bottom) further increasing heterogeneity—at least from a functional perspective. In fact, diverse interactions and stimulations of immune cells result in a broad spectrum of activation states (Fig. 2, right bottom image) [55].

Heterogeneity on the population level is mainly studied in humans, while other aspects of heterogeneity in the immune system are often studied in mice, which serve as a model for humans in both homeostasis and disease. On the population level, it is widely recognized that the immune response may vary significantly among different individuals, depending on genetic background, sex, interaction with pathogenic and commensal microbiota, life style (e.g., exercise, diet, environmental exposure), aging processes and more [56–58]. This variation can already be recognized by cellular composition of individual immune cell types, which can be easily evaluated: Brodin et al. presented a representative summary from two cohorts of the relative frequencies of blood immune cells, measured at the single-cell level via flow cytometry [59]. Despite the fact that the individuals included in these cohorts were classified as healthy, there was a wide range of diversity in the relative frequencies of six major immune cell populations: monocytes, neutral killer cells (NK), B cells, T cells, and specialized T cell subclasses (CD4+ and CD8+ T cells). Similar results were obtained by other studies, affirming that this variability is not cohort specific but rather a universal feature of human populations [60]. While identifying the cellular composition may require single cell techniques, such as flow cytometry or microscopy, for uncovering immune system diversity on the population level, bulk analysis may be sufficient. Several studies show large variation between individuals in gene expression of the total fraction of peripheral blood mononuclear cells (composed of lymphocytes and monocytes), or in the concentrations of proteins in the blood (i.e., soluble molecules related to immune function) [59].

Organ and tissue distribution as well as local niches within the tissue shape immune cells towards different phenotypes and functions. Tissue heterogeneity is described for almost all immune cell types, including γδT cells [61] and macrophages [62]. In this context, resident tissue macrophages have received much focus, and the phenotyping of macrophages among organs and tissues has been extensively studied and reviewed [54]. Resident macrophages are present in almost every organ and tissue in the body under healthy conditions, and their abundance may increase upon insult or tissue remodeling. Performing bulk whole-genome microarrays on populations of macrophages isolated by FACS from different tissues (e.g., liver, lung, and brain), Gautier et al. showed by various analysis methods (e.g., principal component analysis) a high diversity of macrophage phenotypes among tissues [63]. Similar findings were provided later for both transcriptome and epigenomes of tissue macrophages [64]. In addition, even within the same tissue, specific subpopulations of macrophages may exist in different niches. These “niche macrophages” present different features compared to their counterpart resident macrophages [65]. For example, the phenotypic diversity of adipose tissue macrophages, especially in obesity, has been extensively described by microscopic, flow cytometry, and transcriptomic analyses, demonstrating highly diverse phenotypes [66]. A particular subpopulation of lipid-laden macrophages was observed through scRNA-seq and flow cytometry analyses to express a metabolic transcriptional program related to lysosomal and lipid metabolism, secrete high levels of exosomes, and localize mostly in specific structures named crown-like structures, surrounding dead adipocytes and facilitating their clearance from the tissue [67]. Similar evidence for “niche macrophages” was reported in the lung [68], liver [69], and brain [70], emphasizing the contribution of spatial distribution as a determinant for cellular phenotypic heterogeneity.

Among leukocytes of the same class, there is a high degree of heterogeneity, reflected by various states of cellular developmental stage, differentiation, and activation [28]. For example, during T cell development in the thymus, hematopoietic precursors give rise to antigen-responding T cells in a series of differentiation and proliferation events. T cell subsets generated differ in the type of their T cell antigen receptor (i.e., TCR, structured by either αβ or γδ chains), antigenic responsiveness, and effector function. The T cell subsets further differentiate in the thymus and circulation to generate additional cellular phenotypes (such as cytotoxic and helper T cells), expand, and accept different roles (naïve, memory, or effector) [71]. Mousset et al. recently published an update on the flow cytometry-based analysis of the various T cell subtypes using fluorescently labelled antibodies targeting intra- and extracellular proteins [72]. Moreover, further focusing on T cell heterogeneity, genetic variation may also differ among cells. Although immune cells generally do not present genetic variation (though somatic variation may exist [73]), T cells and B cells perform genetic recombination during their development. This process results in highly diverse repertoires of T and B cells, expressing different T cell receptors and antibodies/immunoglobulins (Igs), respectively. Molecularly, this heterogeneity is defined by V(D)J recombination, as it involves a series of complex rearrangements of the sub-regions V, D, and J of the complete gene for the T cell receptor, or Ig for B cells [74]. Additional insertions and deletions of nucleotides in the joining regions between the genes V and D and D and J increases the diversity even further [75]. This process is not purely stochastic, with some regions of the VDJ gene being more frequently used in the recombination process and some receptors presenting higher probability of generation or more commonly shared between different individuals [76]. Revealing the high magnitude of the T cell receptor diversity was enabled by the advancements in high-throughput DNA sequencing technology, allowing deeper sequencing than was previously possible using capillary-based technologies. By adapting such technology, Robins et al. sequenced the genomic DNA of the rearranged regions in the β chain structure of the T cell receptor from millions of T cells, increasing the estimation of the human T cell receptor diversity by fourfolds [77].

The immune response to a certain pathogen or stimulus can be highly heterogeneous within a population of immune cells belonging to the same class or subtype. Diversity in cellular response may rise from intrinsic factors, such as transcriptional burst or cell cycle stage, and extrinsic factors, such as environmental cues and cell-to-cell communication [78]. Heterogeneous responses in immune cells have even been shown for apparently robust average responses measured at the population scale. For instance, Shalek et al. investigated at the single cell level the response of mouse bone-marrow derived dendritic cells to lipopolysaccharide (LPS), a component of Gram-negative bacteria that activates a pro-inflammatory response [79]. Performing scRNA-seq on 18 cells, the authors revealed high variability in gene expression levels among individual cells 4 hours after LPS stimulation, and validated the results by RNA-fluorescence in situ hybridization (RNA-FISH) for 25 selected genes that were within a wide range of expression level. Among the most highly expressed genes, approximately a third presented bimodal expression patterns, characterized by high expression in the majority of the cells, with 3–4 single cells presenting a significantly lower than average expression. These bimodally expressed genes included antiviral and inflammatory response factors. Principal component analysis with the genes showing at least twofold increase upon LPS stimulation revealed two distinct populations, with one group of fifteen cells highly expressing several antiviral genes, while the second group composed of three cells expressed these genes at a much lower level. Further analyzing the transcriptional data, the authors revealed that the two cell subpopulations differed from one another in their developmental stage, as well as in the activities of regulatory circuits.

Overall, the development and application of omics technologies has enabled simultaneous measurements of a plethora of parameters from blood samples, isolated immune cell (sub)populations, or even single cells, and has revealed the extraordinary heterogeneity in the immune system and immunological responses on population and cellular level. Mouse models with a defined genetic background and standardized environmental influences are being used to dissect the heterogeneity on tissue level, and to define mechanisms of heterogeneous immune responses on the cellular level without the genetic and environmental variance. On the other hand, the view on the heterogeneity within the healthy human population is shifting from being a drawback in human studies, requiring larger experimental groups to observe relevant effects, towards being acknowledged as a useful tool to study human biology. With the growing availability of large multi-omics datasets, it has become possible to correlate single features and gene signatures with functional states – an avenue that is poised to be further explored in many more questions and to be applied to other studies.

### Mathematical methods: stochastic and deterministic models to resolve different scales

As George Box puts it, “all models are wrong, but some are useful”. Mathematical methods can be roughly classified into two macro-areas, namely stochastic and deterministic methods. Briefly, stochastic models include a random component that allows the system to evolve in different ways in each simulation, while a deterministic model builds on fixed rules and as long as the parameters and initial conditions are not changed, will always return the same output. Both approaches are equally valid, and the choice between the two mostly depends on the characteristics of the biological questions that are to be addressed by modeling.

Among the most common deterministic models, differential equations describe the variation over time of a variable of interest as a function of other (dynamic) variables and parameters. Ordinary differential equation (ODE) models describe homogeneous systems, and partial differential equations (PDE) are used to account for spatial inhomogeneities. Thanks to the relative simplicity in defining and solving these systems computationally, differential equations are widely used in theoretical biology to model biological systems of different complexity [5]. In general, ODE and PDE models are well suited to describe the dynamics of macroscopic properties of a system. However, differential equation models can easily increase in size in terms of equations and parameters, and it becomes essential that such equations and parameters composing the model are a realistic representation of the physical and biological phenomena. Additionally, when too many parameters have to be estimated (e.g., through fitting) rather than be measured or derived from first principles, the predictive power of the model might become questionable.

Over the past three decades, with the advent of sequencing techniques and the collection of novel insights into gene functions, new approaches to model metabolic regulation in organisms have been developed. Metabolic network models are genome-scale reconstructions of the metabolic pathways of an organism, obtained in semi-automated ways from genome annotation [80]. Once such models are reconstructed (a process per se rather demanding in terms of time, as they still have to be manually curated), they can be analyzed in several ways, including the widely used flux balance analysis (FBA) [81]. Briefly, the metabolic network is represented as a stoichiometric matrix, whose product with the vector of metabolic fluxes gives a system of differential equations. In order to solve such system, a high number of parameters (mostly metabolic reaction rates) has to be estimated [82]. Under the steady-state assumption, the system simplifies and an infinite space of possible solutions is obtained, which can be reduced in size by imposing thermodynamic, biological and physicochemical constraints on the fluxes. An optimization problem is then defined in order to identify one (of many possible) solutions that will maximize (or minimize) an objective function of the fluxes. The arbitrary choice of the objective function makes the FBA method applicable only to biological systems meeting specific conditions. For example, FBA is in general well suited to study microbial ecosystems [83] but still challenging for multicellular organisms [84]. Many extensions of FBA have been proposed to address various shortcomings of the method [85]. In particular, a lot of effort is devoted to integrate high-throughput omics data into the definition of the flux constraints in order to solve the optimization problem in a data-driven, biologically meaningful way [81, 86]. FBA and its variants have been also used to model mammalian cells and their metabolic reprogramming in diseases such as cancer [87]. Although these models still suffer from incomplete functional annotation of the whole cell metabolic pathways, they might greatly improve thanks to the advancements in omics technologies. Metabolic modeling could then also be applied to investigate metabolic regulation in immune cells, taking into account that in order to capture other mechanisms such as cell-to-cell signaling, integration with other methods is needed. Multi-scale modeling and model integration is already actively investigated for microbial ecosystems, and many of these developments could be transferred to other systems [83].

Deterministic methods can describe the average emergent properties of a homogeneous population, much in analogy to experimental bulk measurements. It is still possible to simulate heterogeneous populations as a system of different subpopulations if their emergent behavior can be parameterized. However, in order to capture individual behavior a dramatic reduction of scale is needed at which randomness has to be introduced in the models, especially if the processes determining an individual’s fate are intrinsically stochastic. Monte Carlo methods employ random sampling from probability distributions to computationally simulate a system, hence introducing stochasticity into a deterministic formulation. The Gillespie algorithm is a type of Monte Carlo method that was applied by Dan Gillespie to simulate coupled biochemical reactions [88]. Today, it is probably among the most widely used stochastic methods to model biological systems. This method simulates the temporal evolution of a system as a chain of stochastic events: after the system is initialized, the algorithm proceeds by randomly determining which event (e.g., a reaction) occurs, and the time interval at which it occurs. Time and system state are hence updated and the process continues until some stop conditions are reached. Both Markov and non-Markov processes can—in principle—be modeled with a Gillespie algorithm, and various implementations adapting the original version (computationally expensive) exist.

**Fig. 3:**
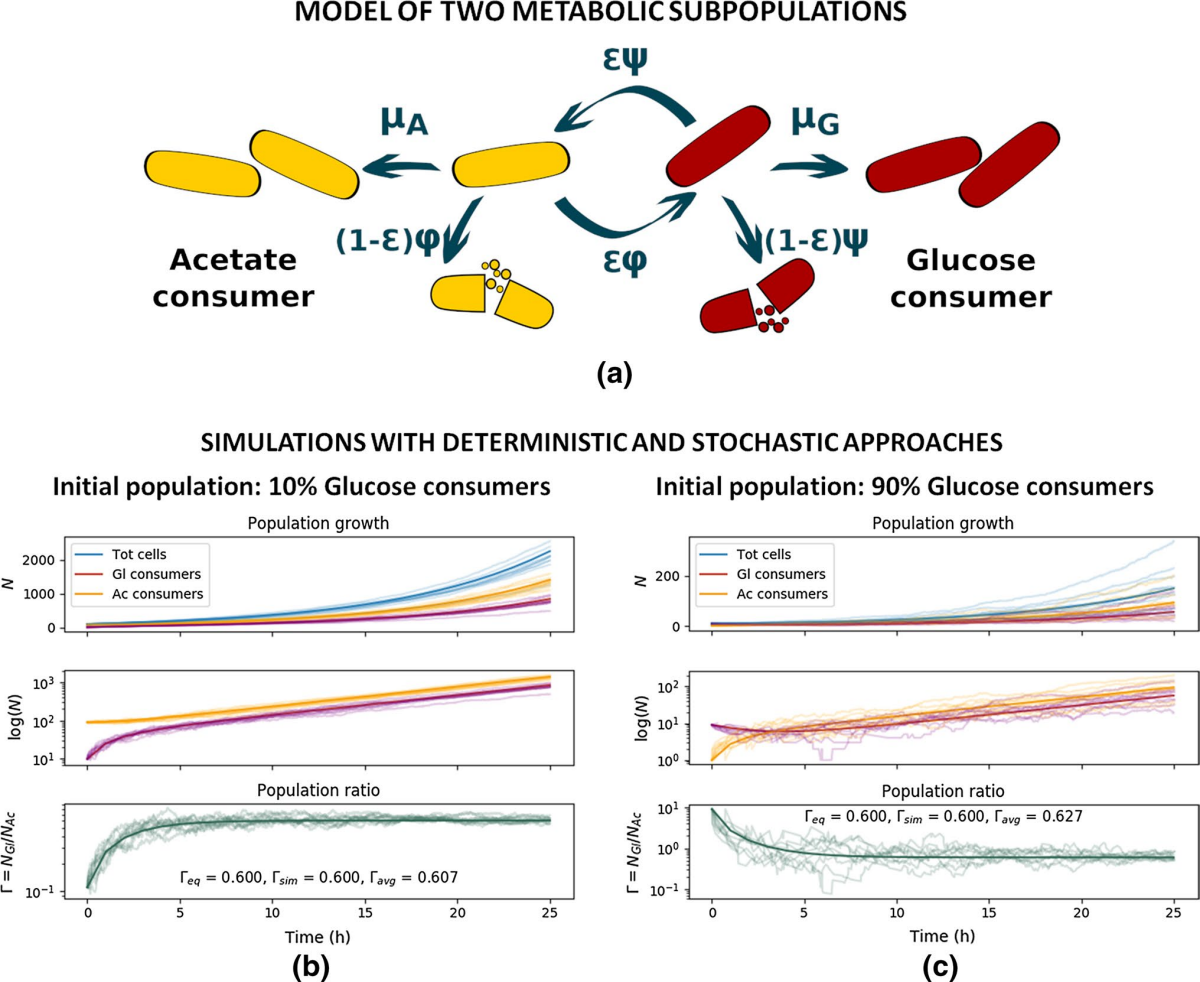
Same system can be described with deterministic and stochastic models. **a** Simplified model of metabolic subpopulations in *E. coli* monocultures. A glucose consumer G (red cells) can grow with growth rate *μ*_G_, or switch to consume acetate with rate *ψ* and efficiency *ε*. In the same way, an acetate consumer A (yellow cells) can grow with growth rate *μ*_A_, or switch to consume glucose with rate *φ* and efficiency *ε*. The cells that are not successful in switching die out. **b, c** Evolution in time of the subpopulations in a constant environment, where only acetate is available as carbon source. The solid lines are the results of the deterministic simulation, and the transparent lines are the results from 10 stochastic simulations. The two top panels show the number of cells for glucose (red) and acetate (yellow) consumer populations, the top panel showing as well the overall observable population size (blue). The bottom panel shows the population ratio *Γ* = *N*_G_/*N*_A_ and reports the equilibrium value *Γ*_eq_ analytically computed, the *Γ*_sim_ value reached by the deterministic model, and the *Γ*_avg_ average value of the stochastic simulations evaluated at the last timepoint. Simulations are run with the parameters: *μ*_G_ = 0 h^−1^; *μ*_A_ = 0.23 h^−1^; *ψ *= *φ* = 0.24 h^−1^, *ε* = 0.9. The initial number of cells are different in the simulation: **b***N*_G_(0) = 10, *N*_A_(0) = 90; **c***N*_G_(0) = 9, *N*_A_(0) = 1

In general, the same system can be modeled with different approaches, and while the different implementations will provide the same general results, the contained information will be different. We illustrate this with an example of monoclonal *Escherichia coli* cultures modeled with an ODE system (deterministic) and with a Gillespie algorithm (stochastic) as a community of two subpopulations. This simplified model (taken from [89], discussed also in the next section) is illustrated in Fig. 3a. In Box 1 the corresponding ODE model is detailed and the equivalent formulation as a Gillespie algorithm is introduced. Both the deterministic and stochastic simulations regard the system as spatially homogeneous, but while the ODE formulation considers time as continuous and the events as fully predictable, the Gillespie algorithm treats the evolution of the system as a unique and non-repeatable random-walk process. In this example of an *E. coli* monoculture in a constant environment allowing continuous exponential growth, the objective of the original model was to investigate the dependence of the subpopulation ratio at equilibrium on the model parameters [89]. The two approaches in this case (and in general) deliver consistent results (Fig. 3b, c), but provide different resolutions: the ODE model provides the average bulk population growth, while each Gillespie simulation represents a possible population trajectory resulting from single cell events. This example comes from a study without specific focus on stochastic metabolic variations in the cell populations and only bulk data were available. Therefore, the deterministic ODE model provided sufficient information with very low computation power requirements. However, if coupled with single cell resolution data, it would make sense to still use deterministic differential equation models only in those systems, where it is possible to group single cells into subpopulations and with the objective to investigate the emergent properties of those subpopulations. Indeed, although it is in principle possible to build deterministic differential equation models at single cell resolution, the dimension of such systems in terms of equations (at least one equation per cell) and parameters (to represent, e.g., cell developmental processes and cell-to-cell interactions) would make the simulations technically demanding and require either the measurement or the inference of thousands or more single cell-specific parameters. Monte Carlo methods such as the corresponding stochastic Gillespie algorithm model would be in such case more efficient, as parameters can be randomly generated from pre-defined probability distributions.

Box 1: Deterministic and stochastic formulation of a simplified subpopulation model of *Escherichia coli* monoculture We consider the model represented in Fig. 3a, with two *E. coli* subpopulations, a glucose (G) consumer and an acetate (A) consumer, where each cell can shift from one population to the other. Assuming that the environment is kept constant, the population dynamics is described by the simplified ODE system:

$$\begin{array}{*{20}c} {\frac{{{\text{d}}N_{\text{A}} }}{{{\text{d}}t}} = \mu_{\text{A}} N_{\text{A}} - \phi N_{\text{A}} + \varepsilon \psi N_{\text{G}} } \\ {\frac{{{\text{d}}N_{\text{G}} }}{{{\text{d}}t}} = \mu_{\text{G}} N_{\text{G}} - \psi N_{\text{G}} + \varepsilon \phi N_{\text{A}} ,} \\ \end{array}$$

where *N*_G_, *μ*_G,_ and *N*_A_, *μ*_A_ are the number of cells and growth rates of glucose and acetate consumers, respectively, *ψ* is the transition rate from glucose to acetate consumer, *φ* is the transition rate from acetate to glucose consumer, and *ε* is a transition efficiency factor (more information in Succurro et al. Supplementary Text 1 [89]).

The equivalent formulation as a Gillespie algorithm [88] comprises the following events:

**Table:** 

Single-cell event	Action	Propensity
*E*_1_: growth of G	$N_{\text{G}} \to N_{\text{G}} + 1$	$\mu_{\text{G}}$
*E*_2_: growth of A	$N_{\text{A}} \to N_{\text{A}} + 1$	$\mu_{\text{A}}$
*E*_3_: successful switch from G to A	$N_{\text{G}} \to N_{\text{G}} - 1; N_{\text{A}} \to N_{\text{A}} + 1$	$\varepsilon \psi$
*E*_4_: unsuccessful switch from G to A	$N_{\text{G}} \to N_{\text{G}} - 1$	$\left( {1 - \varepsilon } \right)\psi$
*E*_5_: successful switch from A to G	$N_{\text{A}} \to N_{\text{A}} - 1; N_{\text{G}} \to N_{\text{G}} + 1$	$\varepsilon \phi$
*E*_6_: unsuccessful switch from A to G	$N_{\text{A}} \to N_{\text{A}} - 1$	$\left( {1 - \varepsilon } \right)\phi$

Figure 3b, c shows the results from the two versions of the model. The 10 stochastic simulations (shown with transparent lines) follow, on average, the deterministic result (solid lines), although a single stochastic simulation might significantly deviate from the deterministic solution. In this example, the models are simulated with the same parameters, changing only the initial number of cells and subpopulation distribution between Fig. 3b and c. As evident from the bottom panel, the equilibrium subpopulation ratio does not depend on the initial conditions, and the variation in this ratio among the different stochastic simulations reduces towards the equilibrium point, although the variation among the absolute number of cells remains high. This exemplifies how, depending on the observable we are interested in, results from a deterministic and a stochastic model might be equivalent or lead to different interpretations.

### Modeling bacterial populations: metabolic regulation and interactions as drivers of heterogeneity

In general, it could be argued that monocellular organisms have been favored objects of study for developing mathematical models: they are rather easy to culture, have relatively simple genomes, and mostly do not organize in complex ordered structures (though fascinating exceptions apply [90]). The first model of bacterial population dynamics dates back to the 1940s, when Monod developed his law for bacterial growth [91]. In his pioneering work, Monod described the different growth phases in bacterial cultures, in particular defining the exponential growth rate and how to measure the lag time. The Monod equation relates the specific growth rate to the concentration of the limiting nutrient similarly to the Michaelis–Menten law for enzyme kinetics, and still today it is used for simple models of bacterial population growth.

Although bacteria present relatively low levels of complexity, being uni-cellular organisms with no tissue or organ infrastructure, microbial systems are still poorly understood and we lack the mechanistic understanding needed to engineer and manipulate the bacterial communities towards desired applications such as medical therapies or biotechnological processes [92]. In natural and artificial ecosystems, the bacterial population mostly interacts through metabolic exchanges, but how a community assemblies and functions exactly, is still unknown. With bottom–up approaches, simplified communities are reconstructed and studied in controlled environments [93]. Such approaches can offer insights into the specific interaction mechanisms around which the experiments were designed, but can only approximate the full scale of heterogeneity within a bacterial community. In an outstanding work, Goldford et al. implemented a top–down approach, combining experiments and theory to study how diverse the assembly of a community under highly controlled experimental conditions can be [94]. First, they collected natural samples of microbial communities from different ecosystems and cultured them in minimal media with a single carbon source, observing high diversity and taxonomical richness. Performing regular culture transfers into fresh media every 2 days and measuring the 16S rRNA profile of the community at each transfer (a total of 12, corresponding to about 84 generations) they assessed the diversity at the species level in terms of exact sequence variants (ESVs) and observed that most ecosystems reached stable community composition around generation number 60. Although taxonomically different at genus and species level, at family level the ecosystems composition appeared strikingly similar across ecosystems. Varying the single carbon source in the minimal media, Goldford et al. obtained different family-level compositions which were highly predictable with a machine learning approach. These results, which were further supported by metagenomics analysis, are consistent with the ecological principle that the environment selects for functional groups that can, however, show high taxonomical heterogeneity. To further investigate niche partitioning in the communities and in particular the hypothesis that co-existence is facilitated through secretion of by-products, the authors developed a consumer-resource model, also known as MacArthur model. A consumer-resource model is a system of ODEs which extends the classic Lotka-Volterra population model by adding the dependence on specific resources beyond the direct intra-species interactions [95]. Goldford et al. generalized the consumer-resource model to include the property of bacteria to actively shape the environment and tested whether simple assumptions on the model could explain the observed experimental results. A minimal environment with one resource and no by-products (reflecting the classic MacArthur model) would indeed lead to competitive exclusion, leaving the ecosystem dominated by a single species with the highest resource consumption rate. Introducing instead rates of by-products secretion and utilization (parameterized for four functionally and metabolically similar groups, corresponding to the four dominant families), the authors could explain the observed diversity in the final population. Indeed, starting with a random assembly of 100 species from the four families, the population converged to the observed family-level structure.

The consumer-resource model is an example of a deterministic and low-complexity model that can still offer insights on emergent properties of large self-assembled communities. In order to study metabolic interactions, FBA and its variants are favored methods which are currently also applied to model microbial communities [83]. However, FBA is also a deterministic method and cannot capture stochastic effects such as noise in gene expression or metabolic differentiation. Instead, the flux solution returned by the FBA result will represent the average metabolic state in a homogeneous population. Combining FBA with Monte Carlo sampling from experimentally measured protein expression distributions, Labhsetwar et al. provided a method to model heterogeneity in a monoclonal population [96]. The authors use a metabolic network model of *E. coli* and randomize the constraint conditions of the FBA problem by drawing the enzyme copy numbers from the distribution of 352 proteins. The enzyme concentration was then used to compute the respective metabolic rates using Michaelis–Menten kinetics. In addition, they used transcriptional regulatory data to further constrain the FBA problem to account for biologically relevant conditions. The process was repeated 1000 times to obtain a population of 1000 *E. coli* cells growing on the same medium but with different metabolic profiles. The predicted growth rates consistently averaged to a value similar to the bulk growth rate, and few genes were identified to account for most of the metabolic variability. However, the usage of significant experimental information to constrain and parameterize the FBA model could be considered equivalent to overfitting. How to integrate high-throughput omics data in order to increase the predictive power of FBA-like models is a very active research topic.

Using a more minimalistic approach, Succurro et al. recently proposed a dynamic FBA model describing the observed diauxie (the metabolic shift from consuming exclusively one preferred carbon source to consuming a different carbon source upon exhaustion of the first) in *E. coli* monocultures as an emergent property of subpopulation distribution [89]. Integrating an ODE system with the *E. coli* metabolic model and using low resolution data (bulk growth curves in three different environmental conditions), the authors showed that a two-population model rather than a one-population model could match simultaneously the three sets of experimental data. In order to parameterize the model without over-constraining the FBA problem, the authors developed a simplified ODE model under the hypothesis that the subpopulations converge to a constant ratio in a constant environment (see also Box 1 and Fig. 3 from previous section). Although the dynamic FBA model was developed for two subpopulations (as two carbon sources were considered), such approach can easily be extended to increase the population metabolic heterogeneity.

### Modeling immune cells: population heterogeneity resulting from cell differentiation events

The immune system presents several additional degrees of complexity compared to the simple prokaryotic uni-cellular bacteria. Nevertheless, we previously argued that abstraction capability is at the root of the power in theoretical approaches, and complexity can be tackled at different scales. Since the work of George Bell on mathematical models of clonal selection and antibody production in the early 1970s, the interest in theoretical approaches in the field of immunology has steadily grown [97]. As the immunology field covers on its own a wide and diverse range of topics, we will focus here on few recent exemplary works modeling macrophages and T cells. The interested reader can refer to additional reviews dedicated to models of the immune system, such as [11] and [98].

A good example of how simple deterministic modeling can aid in getting practical insights into cell-to-cell interactions is the recent work by Li et al. studying the interaction network of tumor-associated macrophages (TAM) and cancer cells [99]. TAM are highly abundant in the tumor microenvironment, and play a significant role in determining tumor progression [100]. Based on data from the literature, Li et al. developed an in silico computational co-culture model of the crosstalk between cells in a tumor microenvironment as an interaction network of cancer cells (epithelial and mesenchymal) and monocytes differentiating into TAM, defined to exist in two activation states, a “tumor-suppressing” and a “tumor-promoting” state. However, more and more evidence points today to a complex biology of TAM which can exhibit a wide spectrum of activation states, in contrast to the old dichotomist view separating them into only two states [55]. Although different activation states would still fall into the two old categories, intermediate states have also been observed, and are not represented in the model by Li et al. The authors built three ODE models including different types of interactions documented in the literature. The modeled network includes interactions such as direct or mediated interconversion, proliferation, inhibition, activation, and because of feedback loops the effective interactions might be counterintuitive. Each model is composed of five equations describing the variation in time of the five variables (the cell types) with 24 parameters estimated from literature data or within biologically reasonable ranges. Li et al. performed then a steady-state analysis for each model to investigate the allowed population states, and a sensitivity analysis to understand and quantify the impact of each parameter on the steady-state results. In this way, the authors could pin-point the most effective ways to eventually target the system in a medical intervention. For example, in the case of the model including interconversion between the two macrophage states assisted by apoptotic epithelial cancer cells, the stable state, where cancer cells go extinct can be achieved by increasing the rate of conversion of mesenchymal cells to epithelial cells and decreasing growth rate of mesenchymal cells. In principle, this kind of analyses (very commonly used to study biochemical networks) could then assist in the design of effective therapeutic strategies. However, in this specific case the model might be hindered by the oversimplification of the biology of the tumor microenvironment. It would be rather critical to define the activation states in an unbiased fashion to model the dynamics of the interactions between tumor cells and macrophages. Indeed, by not considering the full spectrum of activation states of TAM, or at least one “representative” intermediate state, the underlying biology is possibly not sufficiently described. Furthermore, especially in this context it would be ideal to actually combine theoretical approaches with experimental validation to challenge the model predictions and eventually correct the model assumptions.

As previously argued, deterministic models are well suited to describe the emerging behavior of a system, but cannot capture individual-level processes. Upon infection, cytotoxic T cells (CD8+ T cells, which express high extracellular expression of the marker CD8) undergo rapid proliferation and differentiate from naïve cells into short-lived effectors or longer-lived memory T cells through a diversification path that is not yet completely elucidated. Buchholz et al. studied the differentiation route using a method called in vivo fate mapping in mice, by which immune cells with traceable markers from a donor mouse are transplanted into a recipient mouse [101]. After transplanting either 100 CD8+ T cells from eight differently marked donor mice or one single CD8+ T cell from seven donor mice plus 100 CD8+ T cells from the other donor mouse, they tracked the respective unique markers during cell expansion and diversification in the recipient mouse. The authors were thus able to identify the origin of descendant populations of T cells in response to infection and found that on average about 28% of the single cells generate a detectable progeny. Using this information, they built a statistical population model assuming that the number of single cell-derived progenies was binomially distributed. By randomly sampling such distribution, they generated in silico progenies of 100 cells that were consistent with the measured data. They further observed that 12 days after the infection there was a clear distinction between most of the single cell progenies, which showed a low population number (“dwarves” of size 4000), and few descendants that proliferated to populations about 20 times more abundant (“giants”). Overall, the authors measured that about 5% of precursor naïve cells generate more than 50% of the descendants, a trend that they observed also few days earlier after the infection. Being able to distinguish between different diversification states of the single cells based on the markers, Buchholz et al. built stochastic dynamic models of all possible pathways leading from naïve cells to central memory precursor (TCMp), effector memory precursor (TEMp) and effectors (TEF). Out of the 304 models, simulated with a Gillespie algorithm and fitted to the data, the authors found that only two versions of the interconversion network could fit the experimental results. Both these models describe a linear pathway of CD8+ T cell differentiation, from naïve to TCMp to TEMp to TEF, and correctly predicted the phenotypic profile of the cell population at different days after infection. From the stochastic model, the authors could also understand that variability in the population is driven by early events in each single cell progeny. For this reason, they argued that a minimum size of initial progenitors (identified as about 50 naïve cells) is required to ensure robustness of the immune response of CD8+ T cells during infection.

When studying immunological responses, inferring the dynamic of the process over time has been commonly studied by generating snapshots from multiple timepoints. The analysis and interpretation of such data, e.g., to resolve the trajectory of a single cell over time, is rather challenging. For example, measuring population dynamics with a time series of single cell snapshot data might indicate the decrease in the relative proportion of a certain cell type, but such effect might be due to either increase in its death rate, decrease of proliferation rate, or differentiation into other cell types. The concept of developmental trajectories has been used in the past few years to describe cellular development as a transition in transcriptomic states measured with scRNA-seq data. Such trajectories typically captured the dynamic evolution in a “cell state” space metric rather than in real time and, therefore, represented a static description of the system [102]. Fischer et al. recently proposed a mathematical framework that integrates population size data and time series of snapshot experiments into a model capable of quantifying selection pressure, population dynamics, and differentiation effects [103]. With their pseudodynamics framework, the authors describe the cell population dynamics along cellular states of reduced dimension with respect to the original, high-dimensional molecular space. They developed a PDE model to include time-dependence as well as continuous rather than discrete cellular states. The model includes diffusion, drift, and reaction terms representing, respectively, stochastic changes, development-directed changes, and proliferation or death. To parameterize the model through a maximum likelihood method, two input data sets are used: time-resolved single-cell population distributions and time-resolved cell counts. Fischer et al. applied the model to study the process of beta-selection in T cells, which is a developmental checkpoint at which precursor T cells are either selected for further differentiation or undergo cellular apoptosis. Those cells that pass beta-selection and become αβ-T cells then undergo a proliferative burst. Thereafter, selection of productive and elimination of self-reacting T cells occurs through positive and negative selection, respectively. To study this process, the authors used data generated from 19 scRNA-seq thymus samples from mouse embryos at eight different timepoints after fertilization. In this case, the PDE model consisted of two equations coupled at the branching region, where cells can switch from one trajectory to the other. They first confirmed a previously established developmental model, providing, however, higher resolution for the expression profiles along the developmental trajectory. Using pseudodynamics, they identified two points characterized by high drift parameters. There, fast transcriptomic development (corresponding to states of sequential regulation of transcription factors) determines the behavior of individual cells. The authors further identified a developmental checkpoint (saddle point in the developmental potential function) characterized by low drift and stochastic components which was consistent with beta-selection according to the transcription profiles. Afterwards, high reaction terms indicated high birth–death rates, and cells would undergo positive and negative selection. The authors confirmed then the interpretation of the saddle point as beta-selection point by repeating their analysis with knockout mice producing T cells that cannot go beyond beta-selection. This work represents a step forward in the characterization of the Waddington’s landscape for cell development [104] and can be extended to include additional cell state spaces in the future.

## Conclusions and outlook

In this review, we offered a broad overview of how different disciplines and expertise can integrate to advance our understanding of the natural world. In particular, the full potential of technological progress such as single cell sequencing can be exploited only when combined, in parallel, with equivalent developments of algorithms and computing infrastructure for data processing. Furthermore, harmonization of protocols and standards is highly needed in such a fast-paced environment. We then eluted to a cross-disciplinary, fascinating problem in the biological sciences, where such fast-paced progresses already had a major impact: providing high-resolution data to advance our ability to understand population heterogeneity across different scales. Two research fields were cellular heterogeneity has lately addressed particular attention are microbiology and immunology. Despite presenting rather system-specific biological features, they can often be studied with similar approaches, including using mathematical and computational approaches. Indeed, theoretical biology is the art of abstraction, and methods developed for specific systems have the potential to be transferred across disciplines. However, an essential element to develop a meaningful model is to have a sound biological understanding of the different parts composing the system. With the current fast-paced production of knowledge, it becomes particularly crucial to constantly challenge even established views by following the principles of the scientific method itself. When applied to mathematical modeling, this means to use experimental evidence to construct a model, run simulations to predict new behaviors of the system (e.g., response to a perturbation) and obtain new data to challenge the model prediction. A successful integration of theoretical and empirical methods requires, therefore, a significant effort in communication between experimentalists, bioinformaticians and modelers. Overall, the objective of science is to make progress in our understanding of the natural world through discoveries. Having theoretical models at hand to generate hypotheses that would guide experimental design is an extremely valuable resource as already proven in other fields such as physics.
